# Self-Reactive Carbon Dioxide Absorbent with Sodium Carbonate-Based Hydrogel

**DOI:** 10.3390/gels11010078

**Published:** 2025-01-20

**Authors:** Jae Young Kim, Youn Suk Lee

**Affiliations:** Department of Packaging, Yonsei University, Wonju 26493, Republic of Korea; jerrykjy@naver.com

**Keywords:** sodium carbonate-based hydrogel, self-reactive CO_2_ absorbent, faster CO_2_ capture at low temperature

## Abstract

Sodium carbonate is an abundant, low-cost, and low-hazard raw material widely used as a food additive and CO_2_ absorbent in the food industry. However, its application in food packaging is limited because it is used in solid form, either in sachets or as a compounding ingredient in plastics. Solid sodium carbonate requires an external moisture supply for CO_2_ absorption, with its performance dependent on moisture availability. This limitation hinders its commercialization in food packaging applications. We developed a sodium carbonate-based, self-reactive CO_2_ absorbent hydrogel incorporating polyacrylic acid sodium salt (PAAS). This sodium carbonate hydrogel (SCH-PAAS) exhibits self-reactivity, eliminating the need for external moisture, and demonstrates a high CO_2_ absorption capacity. PAAS incorporation facilitates the formation of a porous structure during gel solidification through reactions with CO_2_. Increased PAAS content accelerates CO_2_ absorption rates, particularly under low-temperature conditions (10 °C and 25 °C). Notably, absorption was faster at 10 °C than at 25 °C. The proposed SCH-PAAS exhibits a significantly enhanced absorption performance at low temperatures compared to conventional sodium carbonate-based materials, which exhibit reduced efficiency under such conditions. The increased gas–liquid contact area in SCH-PAAS makes it highly suitable for fresh food packaging applications, particularly under low temperatures.

## 1. Introduction

Sodium carbonate (Na_2_CO_3_) reacts with CO_2_ and moisture to form sodium bicarbonate, as shown in Equation (1) [1]:

Na_2_CO_3_ (*s*) + CO_2_ (*g*) + H_2_O (*g*) → 2NaHCO_3_ (*s*) (1)

Various applications have been developed for CO_2_ absorbents in coal-fired power plants owing to their environmental friendliness, low cost, and easy regeneration of sodium carbonate. They are effective in overcoming drawbacks such as the corrosivity and high regeneration energy of commonly used amine-based absorbents. In recent years, more effective methods have been developed, including an encapsulated Na_2_CO_3_ aqueous sorbent, a Na_2_CO_3_–carbon nanocomposite [2,3].

In the food industry, sodium carbonate is used as a CO_2_ scavenger in active packaging. CO_2_ increases the storage of some foods, whereas in foods such as coffee and kimchi, it causes expansion during distribution [4]. However, the low capacity of CO_2_ absorption of sodium carbonate compared with that of calcium hydroxide (Ca(OH)_2_) makes it unsuitable for field applications. Calcium hydroxide reacts with CO_2_, as shown in Equation (2)

Ca(OH)_2_ (*s*) + CO_2_ (*g*) → CaCO_3_ (*s*) + H_2_O (*l*) (2)

From (1) and (2), the mass-based CO_2_ absorption capacities of sodium carbonate and calcium hydroxide are 9.43 × 10^−3^ mol g^−1^ and 1.35 × 10^−2^ mol g^−1^, respectively [5].

Moreover, the suitable temperature range for the CO_2_ capture of support-free hydrated sodium carbonate powders is 30–50 °C, owing to the low efficiency caused by the synthesis of Na_2_CO_3_·7H_2_O at lower temperatures [6]. Despite several drawbacks, using Na_2_CO_3_ as a CO_2_-absorbent material has several advantages. First, because water is required for chemical reactions, the absorption rate can be controlled by adjusting the moisture content [7]. Second, because the absorption reaction occurs even in aqueous solutions, the concentration can be optimized and the material can be applied in various forms (such as beads, slurries, and capsules) [8,9,10,11,12]. Finally, the reaction product, NaHCO_3_, is a natural deodorant that removes off-odors from food [13]. To enhance its application in food packaging, its lower mass-based CO_2_ absorption capacity at lower temperatures should be improved, especially when compared to the competitors described above. Additionally, its passive–active properties, which rely entirely on the moisture supplied by food, need to be enhanced.

The key to addressing the limitations of Na_2_CO_3_ as a CO_2_ absorbent lies in its interaction with water molecules, particularly those surrounding it. Yuanhao Cai et al. [6] demonstrated that free-flowing hydrated sodium carbonate powders containing 30 wt% water (HSCP-30) achieved a high CO_2_ sorption capacity of 282 mg/g within 60 min and a rapid CO_2_ uptake, with 90% saturation reached in 16 min. They attributed this performance to the role of additional water in enhancing CO_2_ sorption. Similarly, Xiaoyang Shi et al. [14] reported a reversible chemical reaction in Na_2_CO_3_ nanopores driven by water content. Reducing the water molecules in these pores promoted the hydrolysis of CO_3_^2−^ to HCO^3−^ and OH^−^, enabling the development of a nano-structured CO_2_ sorbent that spontaneously binds CO_2_ from ambient air.

One effective method for controlling water content is using superabsorbent polymers, which can be converted into hydrogels. These hydrogels, composed of cross-linked networks of hydrophilic polymers, can absorb up to 400 times their weight in deionized water or 150–300 times their weight in irrigation water. Their cross-linked structure enables the swelling of the three-dimensional network without polymer dissolution [15,16]. Considering these properties, superabsorbent hydrogels are widely used in sanitary products, food packaging materials, water-retaining aids in agriculture, and pharmaceutical drug delivery systems [17].

I have developed a sodium carbonate-based hydrogel (SCH) using superabsorbent polymers to enhance Na_2_CO_3_’s application as a CO_2_ absorbent in food packaging materials. The SCH exhibits self-reactivity and a high CO_2_ absorption capacity, eliminating the need for an external water supply and significantly improving CO_2_ absorption efficiency.

## 2. Results and Discussion

### 2.1. CO_2_ Absorption Capacity

#### 2.1.1. Preparation of Hydrogels

At a temperature of 10 °C, the samples with varying concentrations of polyacrylic acid sodium salt (PAAS) (denoted as SCH-PAAS/X, where X represents the symbolic mass percentage of PAAS in the mixture) to evaluate their CO_2_ absorption capacity are shown in Table 1. The mixing ratio of sodium carbonate to water was fixed at 40:60 wt%, and PAAS was added at concentrations of 0, 0.25, 0.5, 0.75, and 1.0 wt%. Glycerin was added at a concentration of 9.0 wt% as a viscosity modifier, and phenolphthalein was used as a pH indicator.

#### 2.1.2. Determination of CO_2_ Absorption Capacity

To evaluate the CO_2_ absorption capacity of the SCHs, a pressure drop measurement method was employed in a closed chamber corresponding to the absorption of CO_2_ by the material. The results were expressed as carbonation reaction capacity. The carbonation reaction capacity (*R_C_*, mg CO_2_/g Na_2_CO_3_), defined as the amount of CO_2_ absorbed per gram of sodium carbonate, was used to compare the absorption capacities of the hydrogels on an equal basis, as the quantity of loaded Na_2_CO_3_ varied among samples.

**Figure 1:**
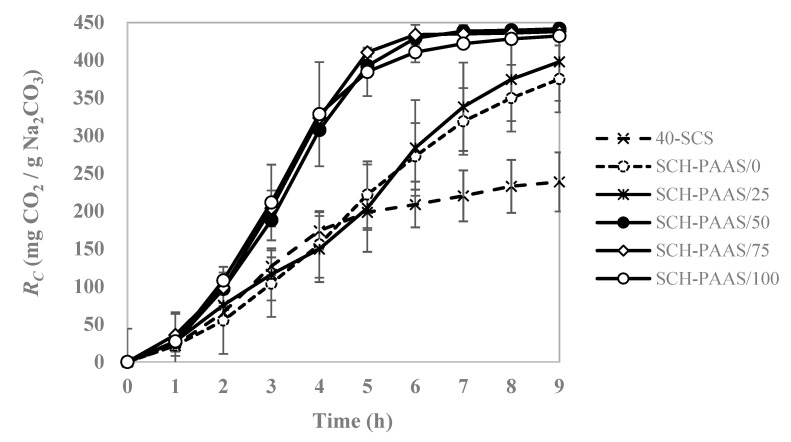
Changes in carbonation reaction capacity (*R_C_*) in different SCH-PAASs as a function of time at 10 °C.

In all the experimental groups where PAAS was added (Figure 1), CO_2_ absorption capacity significantly improved compared to the control groups, which are without the addition of PAAS at 10 °C. From Equation (1), the theoretical *R_C_* value of sodium carbonate was calculated to be 415.23 mg CO_2_/g Na_2_CO_3_. The larger the amount of PAAS, the faster it took to reach the 100% theoretical *R_C_* value at low temperatures. The *R_C_*s of SCH-PAAS/50, 75, and 100 were 429.34, 433.90, and 410.73 mg CO_2_/g Na_2_CO_3_ after 6 h, respectively. There was no significant difference between SCH-PASS/50, 70, and 100. After 6 h of reaction with CO_2_ samples containing more than 0.5 wt%, PAAS exhibited absorption rate was 1.8 times faster than the control and more than twice as fast compared to the 40% sodium carbonate solution.

**Figure 2:**
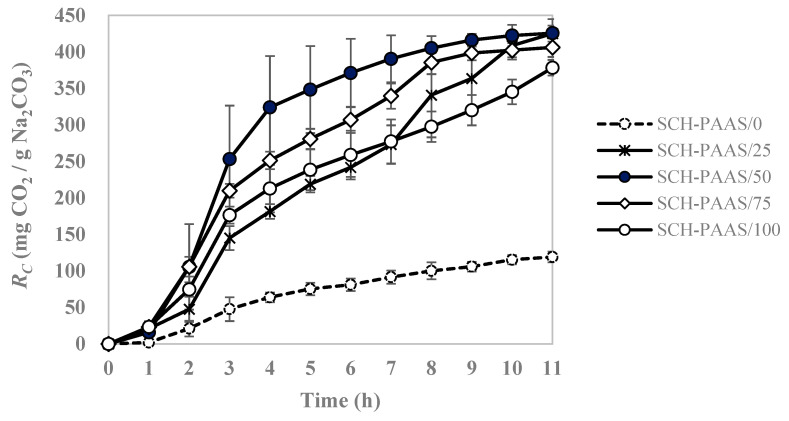
Changes in carbonation reaction capacity (*R_C_*) in different SCH-PAASs as a function of time at 25 °C.

To examine the effect of temperature on the CO_2_ absorption functionality of hydrogel materials, the absorption performance was evaluated at 25 °C. The *R_C_* of SCH-PAAS/50 was 416.17 mg CO_2_/g Na_2_CO_3_ after 9 h in the chamber, reaching 100% of the theoretical carbonation capacity and making it the fastest among the experimental groups, as shown in Figure 2. This performance demonstrated a CO_2_ absorption capacity four times higher than that of the sample without PAAS (SCH-PAAS/0) after 9 h.

The result at a temperature of 25 °C indicates that the CO_2_ absorption capacity increased with the PAAS content up to 0.5 wt%. However, a further increase in PAAS content resulted in a decrease in the CO_2_ absorption capacity. This may be due to the increased gel density with higher PAAS content and the pore-clogging phenomenon caused by the narrowing of pores from the reaction-produced NaHCO_3_ during the reaction with CO_2_ [12].

Based on the experimental results at 25 °C, shown in Table 2, a comparison of the total CO_2_ absorption capacity (*A_C_*, mg CO_2_/g sorbent), defined as the amount of CO_2_ absorbed per 1 g sorbent, revealed that after 9 h, the sample with 0.5 wt% PAAS content absorbed 150.66 mg CO_2_/g sorbent. Samples with 0.25 wt% and 0.75 wt% PAAS content absorbed 132.01 mg CO_2_ and 143.84 mg CO_2_/g sorbent, respectively. Therefore, based on the experiments conducted at 10 °C and 25 °C, the optimal PAAS content for achieving the most effective CO_2_ absorption capacity is evaluated to be 0.5 wt%.

As mentioned above, the reaction time was faster at 10 °C compared to 25 °C because of the increase in CO_2_ solubility with decreasing temperatures. This is attributed to the solubility of CO_2_ in water increasing from 0.149 g CO_2_/100 g water at 25 °C to 0.234 g CO_2_/100 g water at 10 °C [18,19]. The increase in carbon dioxide solubility enhances the concentration of H^+^ and HCO_3_^−^ on the surface Na_2_CO_3_. The kinetics of CO_2_ captured on Na_2_CO_3_ is not controlled by the surface carbonation process but is diffusion-controlled [20,21]. According to Fick’s first law, as shown in the equation below, the diffusion flux (*J*) is proportional to the concentration (*C*). Therefore, the increased carbon dioxide solubility at 10 °C compared to 25 °C is considered to enhance the carbon dioxide absorption rate of the hydrogel.

$$J=-D\frac{\partial C}{\partial x}$$

where *J* is the diffusion flux (mol/m^2^·s), *D* is the diffusion coefficient (m^2^/s), *C* is the concentration, and *χ* is the distance.

Its absorption capacity demonstrates a high performance. The observed CO_2_ absorption capacities of the composite sorbents containing Na_2_CO_3_ were primarily determined by their Na_2_CO_3_ loadings. Theoretically, all the Na_2_CO_3_ on the surface of the sorbents can be used for CO_2_ absorption according to Equation (1) in a humid atmosphere. However, in many studies, the theoretical absorption capacities were not achievable because the active ingredients could not be distributed equally on the surface of the sorbents [11]. This study addresses the issue by demonstrating that PAAS, functioning as a water-lock material, can supply water to Na_2_CO_3_ molecules throughout the gel.

### 2.2. Properties of Hydrogel with CO_2_ Absorption

#### 2.2.1. Phase Change

The carbonation reaction described by Equation (3) consumes hydroxide ions (OH⁻), leading to a decrease in the pH of the hydrogel from the CO_2_ absorption.

OH^−^ + CO_2_ → HCO_3_^−^ (3)

**Figure 3:**
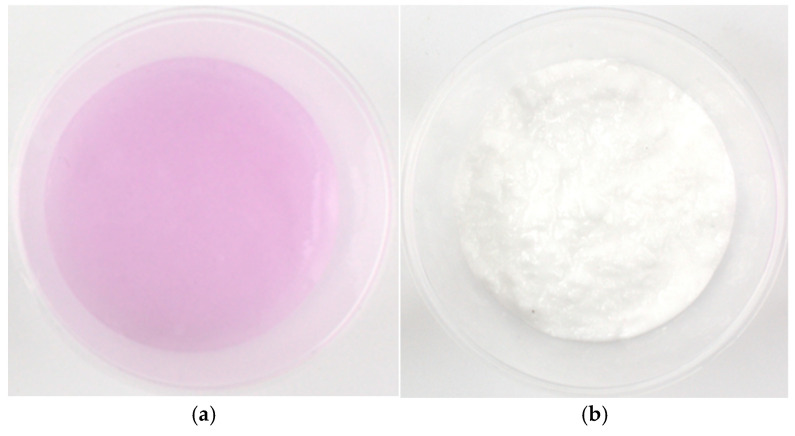
Change in SCH-PAAS/50 color after the reaction with CO_2_ for 6 h at 10 °C: (**a**) before the reaction with CO_2_, (**b**) after the reaction with CO_2_.

To visually assess CO_2_ absorption, we used phenolphthalein as a pH indicator in the preparation of the hydrogel. The pH of the hydrogel before reacting with CO_2_ was measured at 11 (Milwaukee MW102 PRO + 2-in-1), indicating a pink color. After 6 h of reaction with CO_2_, the pH of the hydrogel was neutralized, causing it to turn white because of the colorless nature of the indicator at a neutral pH (Figure 3).

#### 2.2.2. Chemical Change

**Figure 4:**
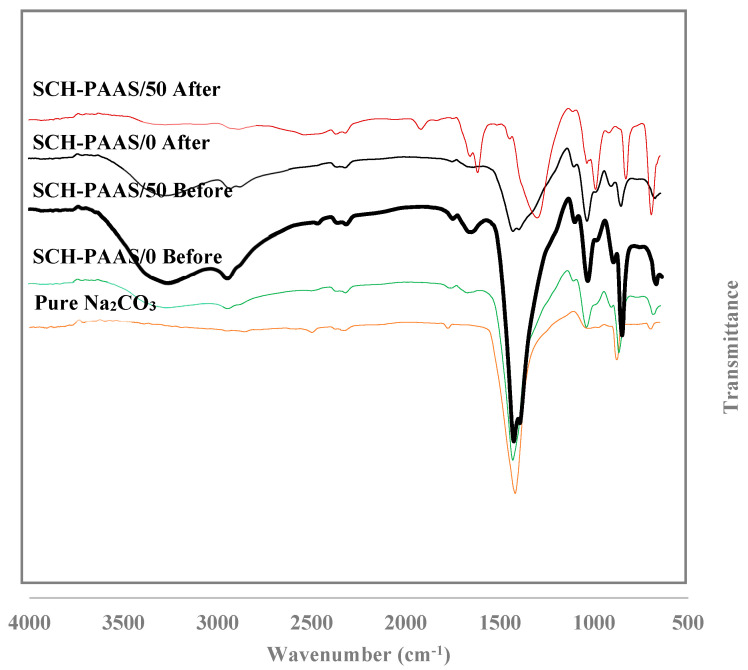
FT−IR of SCH−PAASs before and after the reaction with CO_2_.

**Figure 5:**
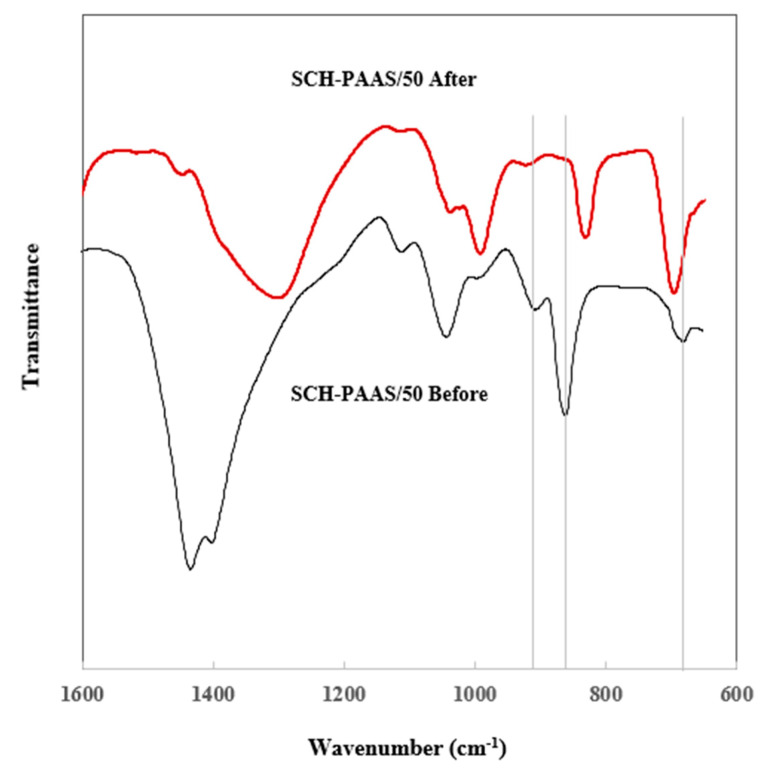
FT-IR of SCH-PAAS/50 before and after the reaction with CO_2_.

Figure 4 shows the FI-IR spectra of pure Na_2_CO_3_, before and after its reaction with the CO_2_ of SCH-PAAS/0 and 50. Joshi et al. [22] used the independent peaks of each substance to quantify Na_2_CO_3_ and NaHCO_3_ in a solid mixture: Na_2_CO_3_ at 1450 cm^−1^, NaHCO_3_ at 1000 and 1923 cm^−1^. All the samples before the reaction exhibit a strong peak characteristic at 1450 cm^−1^ (Na_2_CO_3_) in Figure 5. After the reaction with CO_2_ for 6 h, SCH-PAAS/50 showed two peaks at 1000 and 1923 cm^−1^ (NaHCO_3_) and a significantly decreased peak at 1450 cm^−1^, whereas the peak at 1450 cm^−1^ remained in SCH-PAAS/0, and the two characteristic peaks of NaHCO_3_ did not appear. This indicates that the conversion of NaHCO_3_ from Na_2_CO_3_ in SCH-PAAS/50 was highly effective.

Figure 5 shows peaks at 906, 860, and 679 cm^−1^, which correspond to the monohydrate [23] in SCH-PAAS/50 before the reaction. However, these peaks were not observed after the reaction with CO_2_. During the preparation of the hydrogel, the conditions necessary for the formation of the monohydrate, as described in Equation (4), are as follows: excess water vapor, a low temperature (below 333 K), and an insufficiently high concentration of CO_2_ in the gas phase [24]

Na_2_CO_3_ (*s*) + H_2_O (*g*) ↔ Na_2_CO_3_·H_2_O (*s*) (4)

Na_2_CO_3_·H_2_O (*s*) + CO_2_ (*g*) → 2NaHCO_3_ (*s*) (5)

Thus, when the hydrogel absorbs CO_2_, the reactions shown in Equation (5) are also observed. The presence of this monohydrate may explain the result of rapid CO_2_ absorption at 10 °C compared to 25 °C, because of the increased solubility of CO_2_ as the temperature decreases.

#### 2.2.3. Structure Change

In the 10 °C experiment, samples with more than 0.5 wt% PAAS exhibited the same absorption capacity. However, in the 25 °C experiment, a difference in CO_2_ absorption performance based on the PAAS content was observed. To examine the morphological differences in CO_2_ absorption due to PAAS, a SEM analysis was conducted on the samples with 0 wt% PAAS and the sample with 0.5 wt% PAAS, which showed the fastest CO_2_ absorption at 25 °C.

**Figure 6:**
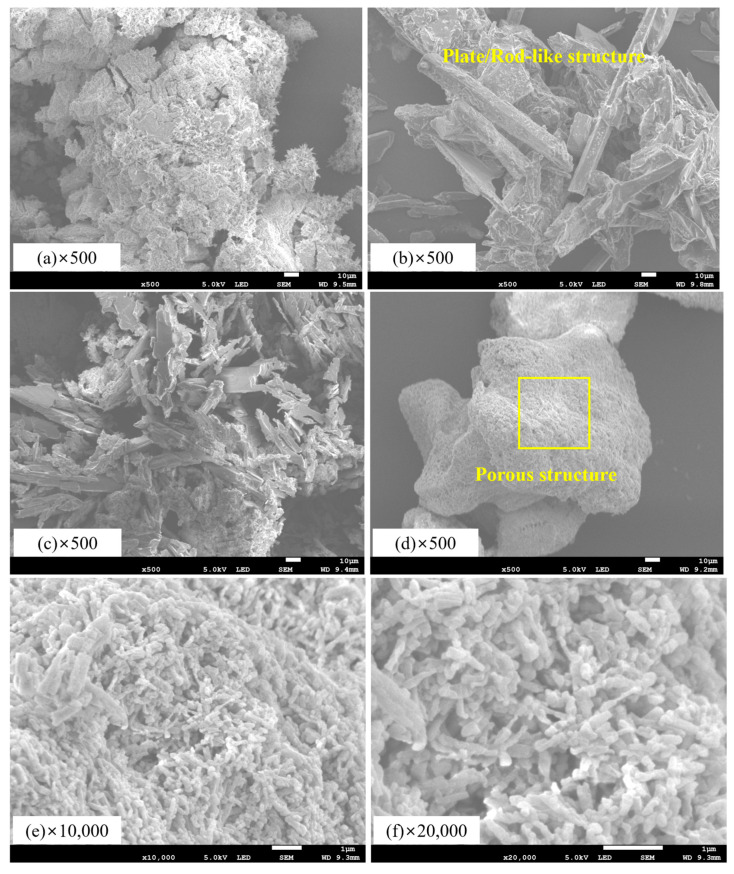
SEM images of SCH with different PAAS ratios: (**a**) before and (**b**) after the reaction with the CO_2_ of SCH-PAAS/0; (**c**) before and (**d**) after the reaction with the CO_2_ of SCH-PAAS/50; (**e**,**f**) enlargement of the special section of (**d**).

Numerous network-shaped pores were observed on the surface of the SCH-PAAS/50 sample containing 0.5 wt% polyacrylic acid salt (Figure 6). After the reaction with CO_2_, numerous porous structures were formed, although the pore sizes decreased to 3.6~5.2 µm compared to those before the reaction. Conversely, on the surface of the SCH-PAAS/0 sample, small and dense Na_2_CO_3_ particles were observed before the reaction. However, after the reaction with CO_2_, large elongated rod-like and plate-shaped NaHCO_3_ solid particles were observed. Luo et al. [24] reported that the macropores on the surface of Na_2_CO_3_ nearly disappeared within 10 min, depending mainly on the higher CO_2_ and H_2_O concentrations, which induced the bicarbonate formation of Na_2_CO_3_.

Therefore, the well-developed network structure of SCH-PAAS/50 was interpreted as having an effect of increasing the gas–liquid contact area.

The addition of PAAS in the preparation of sodium bicarbonate-based hydrogels affects the formation of the gel’s porous structure, particularly enabling the formation of a greater number of porous structures when the gel solidifies through the reaction with CO_2_. This effect occurs because of the uniform and widespread distribution of water molecules and the monohydrate within the cross-linked network structure of PAAS, followed by the conversion of Na_2_CO_3_ to NaHCO_3_ through the carbonation reaction.

## 3. Conclusions

A hydrogel with CO_2_ absorption functionality was developed by adding the superabsorbent polymer PAAS to a sodium carbonate solution. The addition of PAAS overcomes the limitations of traditional sodium carbonate-based CO_2_ absorbent materials, which require an external water supply for activation. As the hydrogel inherently contains water, it reacts immediately with CO_2_, and the inclusion of PAAS enhances its CO_2_ absorption capacity.

Water and sodium carbonate distributed along the PAAS polymer chains prevented the aggregation of solid sodium bicarbonate particles formed as a result of CO_2_ absorption and contributed to the formation of a porous network structure. Owing to this porous structure, CO_2_ was smoothly supplied to the hydrogel, increasing the gas–liquid contact area and resulting in rapid CO_2_ absorption efficiency. This increase in the gas–liquid contact area, combined with the enhanced solubility of CO_2_ as the temperature decreased from 25 °C to 10 °C, produced a synergistic effect, resulting in faster CO_2_ absorption at lower temperatures. Finally, a highly efficient, self-reactive CO_2_ absorbent was developed.

## 4. Materials and Methods

### 4.1. Materials

Sodium carbonate (99.0%, Samchun Chemicals, Pyeongtaek-si, Republic of Korea), polyacrylic acid salt (Sodium polyacrylate, polymerization degree 22,000~70,000, FUJIFILM Wako Pure Chemical Corporation, Osaka, Japan), and glycerin (99.0%, Samchun Chemicals, Pyeongtaek-si, Republic of Korea) were used as reactants. A phenolphthalein indicator was used to visually determine the decrease in pH owing to CO_2_ absorption. Deionized water was used in all the fabrication procedures.

### 4.2. Preparation of the Hydrogels

**Figure 7:**
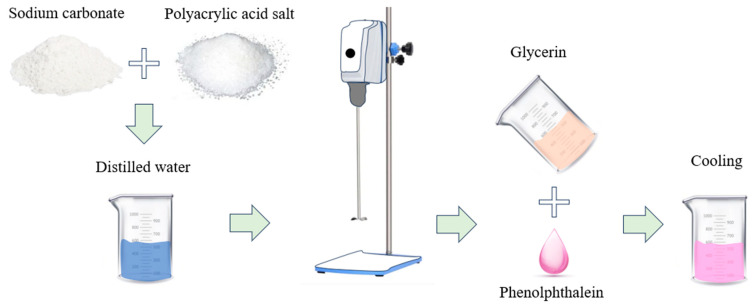
Schematic diagram of the sodium carbonate-based hydrogel preparation.

The mixing ratio of sodium carbonate to water was fixed at 40:60 wt%, and polyacrylic acid sodium salt (PAAS) was added at concentrations of 0, 0.25, 0.5, 0.75, and 1.0 wt%. As shown in Figure 7, the mixture was stirred using an overhead stirrer with a 4-blade impeller for 5 min at 25 °C. Subsequently, 9 wt% glycerin and 3 drops of phenolphthalein indicator were added, and the mixture was stirred for 40 min at 25 °C. Following that, it was cooled in a water bath at 10 °C, while stirring it with a spatula, for 10 min. The hydrogel was stored in bottles in a 25 °C incubator and subsequently used for the experiments.

### 4.3. Properties of the Hydrogels

The morphology of the hydrogels was analyzed using a JSM-7001F (JEOL Ltd., Tokyo, Japan) field-emission scanning electron microscope (FE-SEM). To characterize the chemical structure, all spectra were recorded from 4000 to 650 cm^−1^ using the FT-IR spectrometer (FT/IR-4100, Jasco, Tokyo, Japan), and all measurements were made using 32 scans at 4 cm^−1^ resolution. The samples used for the Fourier-transform infrared (FT-IR) analysis and FE-SEM measurements were freeze-dried for 24 h before use.

### 4.4. CO_2_ Absorption Capacity

**Figure 8:**
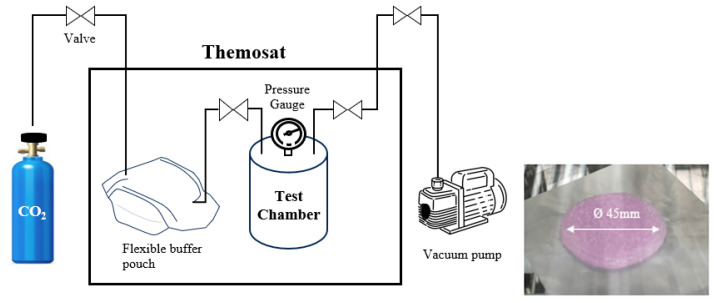
Schematic of pressure drop measurement method to evaluate CO_2_ absorption capacity.

The evaluation of CO_2_ absorption capacity followed the pressure drop method of Vericella et al. [2], which was modified to align with the hydrogel experiment (Figure 8). Initially, a chamber with 5 g of hydrogel was evacuated to −60 kPa using a vacuum pump and subsequently filled with pure CO_2_ gas. The pressure within the chamber was monitored as a function of time using a pressure gauge at 10, 25 °C. In each experiment, a blank chamber without hydrogel was included to monitor the absorption of CO_2_ by residual ambient moisture. The hydrogel used in the absorption experiment was molded with a diameter of 45.0 mm and a thickness of 3.0 mm.

The CO_2_ absorption capacity was calculated using the methods described by Wang et al. [25] and Zhao et al. [10]. From the Ideal Gas Law, the amount of CO_2_ absorption (*n_A_*, mol) in a chamber can be calculated from the pressure drop as follows:

$$n_{A}=\frac{\;\;\;P_{g}\cdot \;V_{C\;\;\;}}{R\;\cdot T}$$

where V*_c_* is the volume of the sample chamber (L), T is absolute temperature (K), R is the gas constant (0.08205 L·atm·K^−1^·mol^−1^), and P*_g_* is the pressure (atm).

The CO_2_ absorption capacity of the hydrogel is expressed as the total CO_2_ absorption capacity (*A_C_*, mg CO_2_/g sorbent), which is defined as the amount of CO_2_ absorbed per 1 g sorbent, and the carbonation reaction capacity (*R_C_*, mg CO_2_/g Na_2_CO_3_), which is defined as the amount of CO_2_ absorbed per 1 g alkali metal carbonate.

$$A_{C}=\frac{1000\cdot n_{A\;}\cdot M_{CO2}}{m}$$

$$R_{C}=\frac{\;\;\;\;A_{C\;\;\;\;}}{\alpha }\;$$

where *n_A_* (mol) is the total amount of CO_2_ absorbed in carbonation (mol), *M*_*CO*2_ is the molecular mass of CO_2_, and *m* and α are the mass of hydrogel and the loaded amount of Na_2_CO_3_ in the sample, respectively.

### 4.5. Statistics Analysis

The data analysis for the experimental results was performed using SPSS software (IBM SPSS Statistics 27, SPSS Inc. Chicago, IL, USA). The data were subjected to an analysis of variance (ANOVA). The statistical significance of differences between the mean values was established at *p* < 0.05, and Duncan’s multiple range test was used for all statistical analyses.

## Figures and Tables

**Table 1:** Sodium carbonate-based hydrogel (SCH) composition with PAAS.

Sample	Materials (wt%)			
	Sodium Carbonate	Water	PAAS	Glycerin
SCH-PAAS/0	36.40	54.60	0.00	9.00
SCH-PAAS/25	36.30	54.45	0.25	
SCH-PAAS/50	36.20	54.30	0.50	
SCH-PAAS/75	36.10	54.15	0.75	
SCH-PAAS/100	36.00	54.00	1.00	

Hydrogel with three droplets of phenolphthalein as pH indicator.

**Table 2:** CO_2_ absorption capacities of the hydrogels after a 9 h reaction in the chamber at 25 °C.

Sample	A_C_ * mg CO_2_/g Sorbent	*R_C_* mg CO_2_/g Na_2_CO_3_	
		Experimental	Theoretical
SCH-PAAS/0	38.51 ± 2.44 ^a^	105.80 ± 6.71 ^a^	415.23
SCH-PAAS/25	132.01 ± 20.99 ^b,c^	363.67 ± 57.81 ^b,c^	
SCH-PAAS/50	150.66 ± 3.16 ^c^	416.17 ± 8.75 ^c^	
SCH-PAAS/75	143.84 ± 4.14 ^c^	398.45 ± 11.47 ^c^	
SCH-PAAS/100	115.27 ± 7.49 ^b^	320.18 ± 20.82 ^b^	

* Values followed by the same letter (a, b, c) are not significantly different (*p* < 0.05) according to Duncan’s multiple range test.

## Data Availability

The original contributions of this study are included in the article. Further inquiries can be directed to the corresponding author.

## References

[1] Liang Y., Harrison D.P. (2004). Carbon dioxide capture using dry sodium-based sorbents. Energy Fuels.

[2] Vericella J.J., Baker S.E., Stolaroff J.K., Duoss E.B., Hardin J.O., Lewicki J., Glogowski E., Floyd W.C., Valdez C.A., Smith W.L. (2015). Encapsulated liquid sorbents for carbon dioxide capture. Nat. Commun..

[3] Nasiman T., Kanoh H. (2018). Preparation of the Na_2_CO_3_-Carbon nanocomposite and its CO_2_ capture. Energy Fuels.

[4] Lee H.-G., Jeong S., Yoo S. (2019). Development of food packaging materials containing calcium hydroxide and porous medium with carbon dioxide-adsorptive function. Food Packag. Shelf Life.

[5] Lee D.S. (2016). Carbon dioxide absorbers for food packaging applications. Trends Food Sci. Technol..

[6] Cai Y., Wang W., Li L., Wang Z., Wang S., Ding H., Zhang Z., Sun L., Wang W. (2018). Effective capture of carbon dioxide using hydrated sodium carbonate powders. Materials.

[7] Park S.-W., Sung D.-H., Choi B., Oh K., Moon K. (2006). Sorption of carbon dioxide onto sodium carbonate. Sep. Sci. Technol..

[8] Yu W., Wang T., Park A.-H.A., Fang M. (2020). Toward sustainable energy and materials: CO_2_ capture using microencapsulated sorbents. Ind. Eng. Chem. Res..

[9] Valluri S., Kawatra S.K. (2021). Use of frothers to improve the absorption efficiency of dilute sodium carbonate slurry for post combustion CO_2_ capture. Fuel Process. Technol..

[10] Zhao C., Chen X., Anthony E.J., Jiang X., Duan L., Wu Y., Dong W., Zhao C. (2013). Capturing CO_2_ in flue gas from fossil fuel-fired powder plants using dry regenerable alkali metal-based sorbent. Prog. Energy Combust. Sci..

[11] Yu F., Wu Y., Zhang W., Cai T., Xu Y., Chen X. (2016). A novel aerogel sodium-based sorbent for low temperature CO_2_ capture. Greenh. Gas. Sci. Technol..

[12] Tuwati A., Fan M., Russell A.G., Wang J., Dacosta H.F.M. (2013). New CO_2_ sorbent synthesized with nanoporous TiO(OH)_2_ and K_2_CO_3_. Energy Fuels.

[13] Jeong S., Yoo S. (2017). Preparation and properties of sodium bicarbonate-incorporated LDPE films with deodorizing function for kimchi packaging. Packag. Technol. Sci..

[14] Shi X., Xiao H., Lackner K.S., Chen X. (2016). Capture CO_2_ from ambient air using nanoconfined ion hydration. Angew. Chem. Int. Ed..

[15] Tomar R.S., Gupta I., Singhal R., Nagpal A.K. (2007). Synthesis of poly (acrylamide-co-acrylic acid) based superabsorbent hydrogels: Study of network parameters and swelling behavior. Polym. Plast. Technol. Eng..

[16] Cannazza G., Cataldo A., De Benedetto E., Demitri C., Madaghiele M., Sannino A. (2014). Experimental assessment of the use of a novel superabsorbent polymer (SAP) for the optimization of water consumption in agricultural irrigation process. Water.

[17] Kim J.S., Kim D.H., Lee Y.S. (2021). The influence of monomer composition and surface-crosslinking condition on biodegradation and gel strength of super absorbent polymer. Polymer.

[18] Dodds W.S., Stutzman L.F., Sollami B.J. (1956). Carbon dioxide solubility in water. Ind. Eng. Chem. Chem. Eng. Data Ser..

[19] Lee S.-W., Chae S., Bang J.-H. (2020). Synthesis of Na compounds from sodium concentrated solution using carbonation and cryo-crystallization. J. Korean Inst. Resour. Recycl..

[20] Cai T., Johnson J.K., Wu Y., Chen X. (2020). Toward understanding the kinetics of CO_2_ capture on sodium carbonate. ACS Appl. Mater. Interface.

[21] Cai T., Chen X., Johnson J.K., Wu Y., Ma J., Liu D., Liang C. (2020). Understanding and improving the kinetics of bulk carbonation on sodium carbonate. J. Phys. Chem. C.

[22] Joshi S., Kalyanasundaram S., Balasubramanian V. (2013). Quantitative analysis of sodium carbonate and sodium bicarbonate in solid mixtures using fourier transform infrared spectroscopy. Appl. Spectrosc..

[23] Rodriquez-Mosqudea R., Bramer E.A., Brem G. (2018). CO_2_ capture from ambient air using hydrated Na_2_CO_3_ supported on activated carbon honeycombs with application to CO_2_ enrichment in greenhouse. Chem. Eng. Sci..

[24] Luo H., Kanoh H. (2017). Fundamental in CO_2_ capture of Na_2_CO_3_ under a moist condition. J. Energy Chem..

[25] Wang H.J., Jo Y.H., An D.S., Rhim J.-W., Lee D.S. (2015). Properties of agar-based CO_2_ absorption film containing Na_2_CO_3_ as active compound. Food Packag. Shelf Life.

